# Impact of Homologous Recombination on the Evolution of Prokaryotic Core Genomes

**DOI:** 10.1128/mBio.02494-18

**Published:** 2019-01-22

**Authors:** Pedro González-Torres, Francisco Rodríguez-Mateos, Josefa Antón, Toni Gabaldón

**Affiliations:** a Department of Physiology, Genetics, and Microbiology, University of Alicante, Alicante, Spain; b Bioinformatics and Genomics Program, Centre for Genomic Regulation (CRG), Barcelona, Spain; c Departament de Ciències Experimentals i de la Salut, Universitat Pompeu Fabra (UPF), Barcelona, Spain; d Departamento de Matemática Aplicada, University of Alicante, Alicante, Spain; e Multidisciplinary Institute of Environmental Studies Ramon Margalef, University of Alicante, Alicante, Spain; f Institució Catalana de Recerca i Estudis Avançats (ICREA), Barcelona, Spain; Duke University

**Keywords:** comparative genomics, core genomes, genome evolution, intraspecific diversity, homologous recombination

## Abstract

Homologous recombination (HR) enables the exchange of genetic material between and within species. Recent studies suggest that this process plays a major role in the microevolution of microbial genomes, contributing to core genome homogenization and to the maintenance of cohesive population structures. However, we still have a very poor understanding of the possible adaptive roles of intraspecific HR and of the factors that determine its differential impact across clades and lifestyles. Here we used a unified methodological framework to assess HR in 338 complete genomes from 54 phylogenetically diverse and representative prokaryotic species, encompassing different lifestyles and a broad phylogenetic distribution. Our results indicate that lifestyle and presence of restriction-modification (RM) machineries are among the main factors shaping HR patterns, with symbionts and intracellular pathogens having the lowest HR levels. Similarly, the size of exchanged genomic fragments correlated with the presence of RM and competence machineries. Finally, genes exchanged by HR showed functional enrichments which could be related to adaptations to different environments and ecological strategies. Taken together, our results clarify the factors underlying HR impact and suggest important adaptive roles of genes exchanged through this mechanism. Our results also revealed that the extent of genetic exchange correlated with lifestyle and some genomic features. Moreover, the genes in exchanged regions were enriched for functions that reflected specific adaptations, supporting identification of HR as one of the main evolutionary mechanisms shaping prokaryotic core genomes.

## Abstract (precis)

Microbial populations exchange genetic material through a process called homologous recombination. Although this process has been studied in particular organisms, we lack an understanding of its differential impact over the genome and across microbes with different life-styles. We used a common analytical framework to assess this process in a representative set of microorganisms. Our results uncovered important trends. First, microbes with different lifestyles are differentially impacted, with endosymbionts and obligate pathogens being those less prone to undergo this process. Second, certain genetic elements such as restriction-modification systems seem to be associated with higher rates of recombination. Most importantly, recombined genomes show the footprints of natural selection in which recombined regions preferentially contain genes that can be related to specific ecological adaptations. Taken together, our results clarify the relative contributions of factors modulating homologous recombination and show evidence for a clear a role of this process in shaping microbial genomes and driving ecological adaptations.

## INTRODUCTION

Gene repertoires in prokaryotic genomes can be divided into the core genome, comprising genes ubiquitously present in all strains of a species, and the accessory (or flexible) genome, comprising genes whose presence is variable within a species (1, 2). Evolutionary analyses of prokaryotic genomes have revealed that changes in the accessory genome are often associated with horizontal gene transfer (HGT) and site-specific recombination involving mobile genetic elements, whereas changes in the core genome generally involve vertical transmission and homologous recombination (HR) (3, 4). Furthermore, recent studies revealed that prokaryotic populations are structured in the form of cohesive clusters, each composed of strains with genomic similarity levels above a given threshold, which are separated from other clusters by much larger genetic distances (5, 6). This phenomenon has been observed in several taxonomic groups and in diverse environments (5, 7–9). However, what mechanisms generate these clusters and what evolutionary forces drive genetic cohesion in prokaryotic populations are still not fully understood. In this regard, two main hypotheses have been proposed: (i) the neutral model (10), which highlights the role of HR as a passive, neutral mechanism driven solely by genetic divergence, and (ii) the ecotype theory (11, 12), which emphasizes the role of natural selection and/or genetic drift in maintaining groups with similar ecological features. Note that the two models are not mutually exclusive but rather could act synergistically, with the relative contributions of the models differing across environments or clades (5). Besides the issue of what evolutionary forces could create cohesive population clusters, many other issues remain open. For instance, it is as yet unknown whether HR occurs indiscriminately across the whole genome or whether selection shapes HR patterns. In addition, we still have only a very sparse picture of the impact of HR and how this varies across phylogenetic groups or lifestyles. To address such issues, broad analyses that cover diverse clades and lifestyles under the same analytical framework are needed (1, 13–15). However, genomic analyses focused on intraspecific HR processes are scarce due to technical limitations (16, 17). In addition, most studies have been based on multilocus sequence analysis (MLSA) or considered different methodologies (13, 16) or few species or focused on individual genomic factors (18–20). Here we set out to assess intraspecific genomic exchange occurring through HR on a taxonomically broad data set and to perform a comprehensive analysis of a wide range of functional, ecological, and genomic factors. To our knowledge, this was the first such broad study to use a common methodological framework and to consider a broad range of potentially relevant genomic factors.

## RESULTS

### Quantification of homologous recombination.

**FIG 1:**
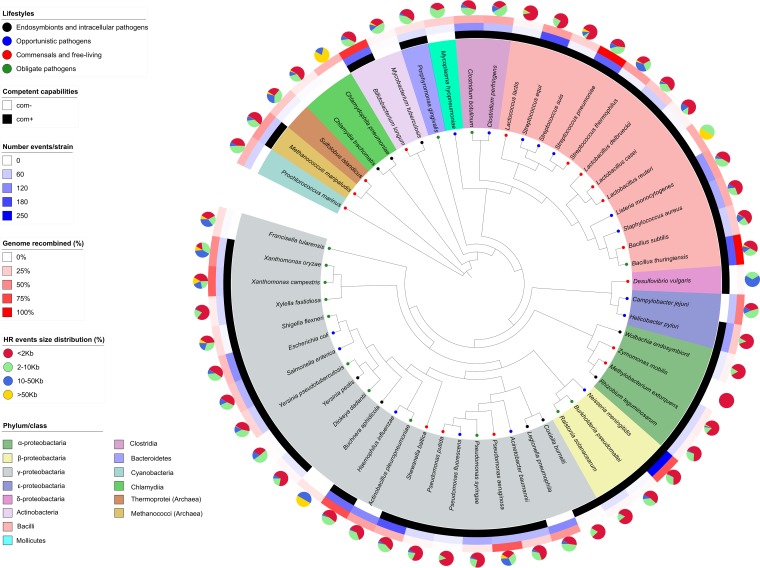
Data set composition. A 16S rRNA phylogenetic tree of the 54 prokaryotic species included in this study is shown. The tree was drawn using itool (https://itol.embl.de/). The innermost circle layer shows the species nape and associated clade. Analysis of HR events was performed. The innermost layer indicates the competence capability. The second (i.e., contiguous) layer, third layer, and fourth layer correspond to the number of HR events per strain, the proportion (%) of recombined genome, and the size distribution (%) of HR events.

We retrieved 338 genomes belonging to 54 bacterial and archaeal species—as defined by the 16S-divergence criterion (21, 22)—spanning a wide range of taxa, environments, and lifestyles—as defined by previous studies (13, 16) (see Materials and Methods) (Fig. 1; see also Table S1A and B in the supplemental material). To enable unbiased comparisons, we used a common methodology to reannotate each genome and quantify parameters based on genomic composition, within-species sequence variation, ecological specialization, and other factors for which a role has been proposed or presumed but whose relationship with HR remains poorly assessed (see Materials and Methods and Table S1B). As the main focus of our study was the comparison across clades and lifestyles with the aim of identifying trends in functional, ecological, and genomic factors related to HR, we prioritized a broader taxonomic sampling over a high number of strains per species. Finally, the sampling set was similar in size and taxonomic distribution to that used in recent studies focused on interspecies exchanges (19).

10.1128/mBio.02494-18.6 TABLE S1 (A) List of the bacterial and archaeal species (corresponding to 338 complete genomes) analysed in this study and 5 controls groups (*). Taxonomic and genomic information is provided on genomic data: genome size (Gs), gene count (Gc), number of scaffolds (Sc), number of CRISPR-Cas systems (CRISPR), GC content (GC), number of CDS (CDS), proportion of pseudogenes (Pseud.), positional orthologs (Ort), proportion of genes transferred horizontally (HGT), and gene annotation: COG, KOG, Pfam, TIGR, Interproscan, TIGRfam, and Enzyme. (B) Main ecological traits, genomic variables, and characteristics and distribution of the recombination events for 54 species and 5 control groups (*) considered in this study. (C) Review of previous studies for different bacterial and archaeal species based on MLSA (white and grey) or whole genomes (orange) in which recombination relative to point mutation (r/m) ratio was evaluated based on a limited and variable number of loci. Highlighted in grey the subset of MLSA studies that estimated r/m using ClonalFrame. (D) Impact and distribution of homologous recombination (r/m and events/strain) for 54 species and 5 control groups (*) considered in this study. (E) Fisher's Exact test results for the most abundant GO terms in COG J, COG L, COG K, COG M, COG N, COG O, COG T, COG V, COG P, and COG G categories among 54 species. In red those terms down-represented and in green over-represented ones once FDR (false discovery rate) correction was performed. Download Table S1, XLS file, 0.4 MB. Copyright © 2019 González-Torres et al. 2019 González-Torres et al. This content is distributed under the terms of the Creative Commons Attribution 4.0 International license.

We next inferred HR events and related parameters for each species by scanning their aligned genomes with a pipeline that combines several HR detection algorithms and selects the events consistently predicted by several methods (see Materials and Methods and Table S1B). This pipeline comprises state-of-the-art methodologies used in recent analyses (19, 23) of HR in microbial genomes, such as RDP4 v4.15 (24, 25) and ClonalFrame 1.2v (26, 27). In brief, these methodologies use a sliding-window approach for the detection of phylogenetically incongruent regions from genome alignments. Different methods implemented in these programs differ in their specific parameters, algorithms, and tests for significance. Therefore, we opted for a conservative approach that selected only those regions that were positive in at least three of the five methods used. In total, we detected 16,300 HR events that were distributed nonhomogenously across the analyzed species (Fig. 1). We found no significant correlation between the number of strains sampled per species and the number of events detected per strain (*P* < 0.05, *r^2^* = 0.048 [Pearson]) or the percentage of recombined genome (*P* < 0.05, *r^2^* = 0.031 [Pearson]), indicating that differences in sample size did not affect our results regarding differences between species.

**FIG 2:**
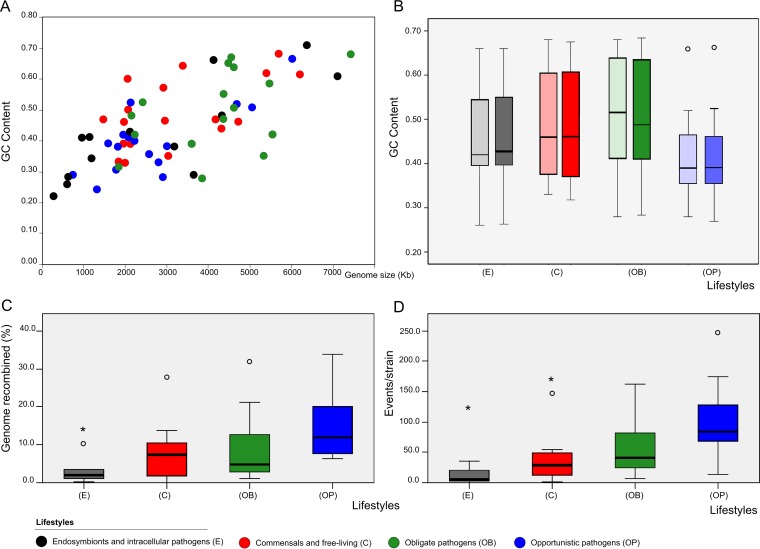
HR characteristics and lifestyle effect. Four lifestyles are represented in all the figures by the same color code: black, endosymbionts and intracellular pathogens; blue, opportunistic pathogens; red, commensal and free-living pathogens; green, obligate pathogens (green). (A) GC content among 54 species included in this study distributed in 4 lifestyles. (B) Box plot comparing average levels of GC content in recombinant events (solid color; right paired boxes) and whole genomes (grayed-out color; left paired boxes). (C and D) HR distribution (events/strain) (C) and proportion of genome recombined (D) based on lifestyle distributions (both *P* < 0.05 [Kruskal-Wallis and Jonkheere-Tepstra tests]).

With some exceptions, and for the species evaluated in previous studies, our estimates were congruent with those reported based on multilocus sequence analysis (MLSA) (Table S1C) or complete genomes (Table S1D), where differences with MLSA may be attributable to the low number of genes typically considered in that methodology (13, 28, 29). The detected HR events differed in the length of the recombined region (i.e. size) (Fig. 1; see also Fig. S2 in the supplemental material), which ranged from 0.1 kb to more than 200 kb. Although only 11% (1,799) of the sequences corresponding to the detected events were longer than 10 kb and 4.5% (75) longer than 80 kb (Fig. S1 [additional file 2]), the proportion of events with large sequences was considerable in some species, in accordance with previous studies (Fig. 1; see also Table S1C) (30–32). In contrast to what had been suggested in previous studies (18) and to what is typically observed in eukaryotes, we did not find significant differences in GC content between regions affected by HR events and the rest of the genome (Fig. 2; see also Table S1B).

10.1128/mBio.02494-18.1 FIG S1 HR event fragment size and gene content distribution. Frequency histograms display the distributions of recombination events amongst the 54 studied species in terms of (A) homologous recombination event size (in kilobases) and (B) gene content. The majority of events involved fewer than 3 kb and fewer than 8 genes. Download FIG S1, TIF file, 0.6 MB. Copyright © 2019 González-Torres et al. 2019 González-Torres et al. This content is distributed under the terms of the Creative Commons Attribution 4.0 International license.

10.1128/mBio.02494-18.2 FIG S2 Functional relation between content of Chi sequences and HR. Data represent correlations of Chi sequence density and HR events/strain (*r*^2^ = 0.57) among seven species with previously described Chi sequences. Download FIG S2, TIF file, 0.4 MB. Copyright © 2019 González-Torres et al. 2019 González-Torres et al. This content is distributed under the terms of the Creative Commons Attribution 4.0 International license.

### Influence of phylogeny and lifestyle.

The effect of the phylogenetic distance on the incidence of recombination has been widely discussed (3, 13, 16). Most studies suggest an inverse relationship between HR rate and sequence divergence (3, 10, 33). In agreement with this, we observed very low recombination rates in the control groups, which comprised genomes from different species of the same genus (Table S1A and D). Previous studies had also suggested that recombination rates may be similar between closely related species (Table S1C), perhaps due to the existence of evolutionary constraints at the genus level (13, 16). However, these studies included only two species that shared lifestyle and therefore did not control for this factor. We tested this hypothesis in our data set, focusing on seven genera comprising several species, among which four comprised different lifestyles (Table S1B). We observed large differences in recombination levels within many of the genera considered. An illustrative example is the genus *Yersinia*, which includes the intracellular human pathogen Y. pestis and the opportunistic pathogen Y. pseudotuberculosis; the latter species showed a much higher recombination/mutation (r/m) rate and 10 times more HR events per strain. Similar trends were observed in the genera *Streptococcus* and *Pseudomonas*, in which HR levels were lower in the free-living species than in their pathogenic relatives. Taken together, our data suggest that phylogenetically closely related prokaryotic species with different lifestyles could present contrasting recombination levels, pointing to an influence of lifestyle in HR patterns.

To gain further insights into the relationship between recombination and lifestyle, we compared HR levels among the members of the four broad lifestyle groups included in our data set (Fig. 2). This lifestyle classification was taken from previous MLSA studies (13, 16), and species were assigned based on the JGI (Joint Genomics Institute) database metadata for each strain. Our results revealed significant differences across lifestyles (*P* < 0.05 [Kruskal-Wallis and Jonkheere-Tepstra tests]). The class containing “endosymbionts and intracellular pathogens” showed the lowest HR levels, followed by “commensals and free-living,” and “obligate pathogens” and with “opportunistic pathogens” showed the highest levels. Of note, this relative order corresponds to those reported from studies for interspecific HGT (34, 35), suggesting that similar constraints may act at the intra- and interspecies levels. Despite this trend, HR levels ranged widely within each lifestyle class (Fig. 1 and 2; see also Table S1D).

### Genomic variables related to ecological specialization and intraspecific DNA exchange.

We next considered different genomic variables that have been found to be related to processes of adaptation and ecological specialization. These include homologous repair systems and competence capabilities as well as defense systems such as toxin-antitoxin, abortive infection (36), restriction modification (RM) (19), and clustered regularly interspaced short palindromic repeat (CRISPR)-Cas (37) systems. We noted that the observed HR trend across lifestyles parallels that observed previously for the content of *rec* genes and the same four lifestyles (38). In this regard, it has been hypothesized that the abundance of Chi sequences (*rec* gene target) should correlate with HR levels (13). We tested this using strains of seven pathogenic species for which Chi patterns were available. Our results confirmed a positive correlation of Chi densities (13) and HR prevalence (Fig. S2). This finding is reinforced by the fact that these species are related only distantly and that their respective Chi sequences are thought to have originated independently (39). This strong coevolution supports the idea of a functional link between *recA* and HR, through the recognition of the Chi-RecBCD complex in Gram-negative bacteria and of Chi-AddAB in Gram-positive bacteria, which act as substrates for the homologous pairing by RecA protein (40, 41).

**FIG 3:**
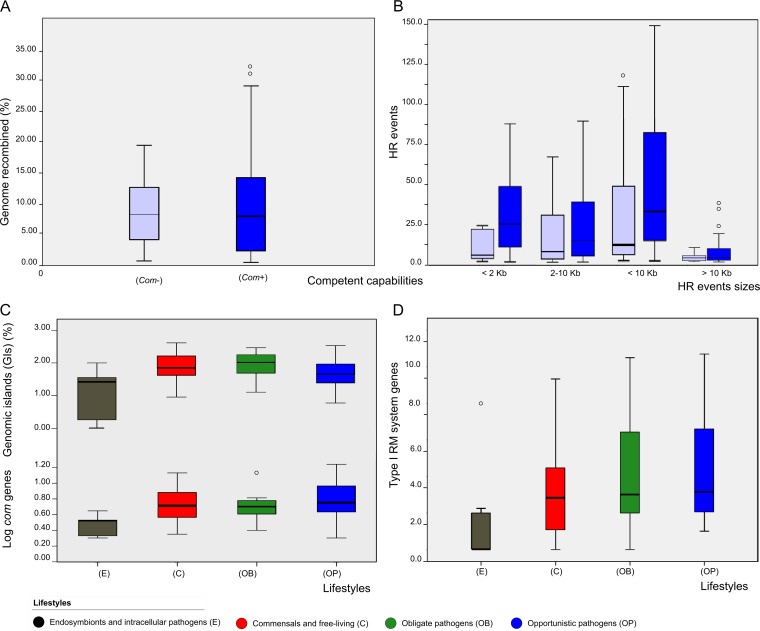
Effect of genomic variables on HR distribution. (A) Proportion (%) of genome recombined based on competence capabilities (*Com*+/*Com*−). (B) HR event fragment size distribution based on competence capabilities: competent (*Com*+) (solid color, right paired boxes) and noncompetent (*Com*−) (grayed out color, left paired boxes). (C and D) Genomic island (GI) distributions (%), *com* gene content (C), and type I restriction modification system (RM-I) gene content distribution (D) based on the different lifestyles considered. Four lifestyles are represented with the following color code: black, endosymbionts and intracellular pathogens; blue, opportunistic pathogens; red, commensal and free-living pathogens; green, obligate pathogens (green).

Besides their essential role in the maintenance of DNA integrity by means of HR, *rec* genes participate in the integration of DNA acquired by conjugation and transformation (3, 42, 43). We explored relationships between genetic acquisition mechanisms and HR levels by classifying species into qualitative competent and noncompetent groups on the basis of metadata available in the Integrative Microbial Genomes (IMG) database and of the presence of the *com* regulon genes (44). Although the fractions of recombined genome were similar in the two groups (*P* > 0.05 [Jonckheere-Tepstra test]), the number of events per strain was significantly larger in competent species, especially when events associated with shorter sequences were considered (Fig. 3) (*P* < 0.05 and *r^2^* = 0.317 for <10-kb linear correlations; *P* < 0.01 and *r^2^* = 0.414 for <2-kb linear correlations). Furthermore, we found significant correlations between the number of short event fragments and the abundance of transposable elements (*P* < 0.05 and *r*^2^ = 0.72 [linear correlation]) and between *com* gene content and transposable elements (*p* < 0.05 *r*^2^ = 0.37, linear correlation). Taken together, these results point to a role of competence in the exchange of short fragments, while conjugative processes may largely be involved in the exchange of longer (>10-kb) fragments, as previously suggested from isolated cases (3, 16). Finally, we observed a significant positive correlation of the number of short event fragments (2 to 10 kb) with both *com* gene content and genomic islands (GIs) (*P* < 0.05, *r*^2^ = 0.43 and *r*^2^ = 0.40 [Spearman’s rho]), which are considered hot spots favoring both HR and HGT exchanges (45) and which were more abundant in pathogens and free-living species (Fig. 3).

Defense systems such as restriction modification (RM) or CRISPR-Cas systems act as barriers modulating DNA acquisition and recombination fluxes (19). We detected significant differences in RM gene content across lifestyles (*P* = 0.001 to <0.05 [Kruskal-Wallis and Jonkheere-Tepstra tests]), even in comparing different RM systems (type I to type III) individually (*P* < 0.05) (Fig. 3; see also Fig. S3). This trend parallels those reported for *rec* genes and HR events among lifestyles such that opportunistic pathogens and free-living species show higher values. Furthermore, we observed a significant positive correlation between the number of events per strain and the content of type I and II RM systems (*P* < 0.05, *r*^2^ = 0.3 and *r*^2^ = 0.43, respectively [Spearman’s rho]). Taken together, these results indicate that species encoding more RM systems tend to acquire more genetic material by HR. This observation is compatible with the role of RM systems generating DNA recombinogenic extremes that may promote HR (46), although it has been observed that intraspecific exchanges are limited between strains coding for different RM type systems (47). In this regard, higher diversities of CRISPR-Cas system genes tended to be associated with lower HR levels (Fig. S4). This suggests that intraspecific diversity in CRISPR-Cas systems, which generally act against heterologous sequences, may as well affect homologous sequences exchanged by conjugation or transformation. Of note, a possible form of coevolution between competence genes and CRISPR-Cas systems has been recently suggested in which loss of competence is followed by loss of CRISPR-Cas systems (48).

10.1128/mBio.02494-18.3 FIG S3 Effect of genomic variables on HR distribution. Data represent type II restriction modification system (RM-II) (A) and type III restriction modification system (RM-III) (B) gene content across lifestyles. Four lifestyles are represented with the following color code:black, endosymbionts and intracellular pathogens; blue, opportunistic pathogens; red, commensal and free-living pathogens; green, obligate pathogens. Download FIG S3, TIF file, 0.3 MB. Copyright © 2019 González-Torres et al. 2019 González-Torres et al. This content is distributed under the terms of the Creative Commons Attribution 4.0 International license.

10.1128/mBio.02494-18.4 FIG S4 Effect of CRISPR-Cas intraspecific diversity on HR. Relationships between the proportions (%) of recombinant genomes and numbers of different CRISPR-Cas system types in genomes are represented. The red line represents the result of the adjustment regression (90th percentile quantile) conducted using the statistical package R Quantreg and the blue line the lineal setting of both variables transformed by logarithm. The figure shows that an increase in the CRISPR-Cas system limited the amount of recombined genome. The legend shows the slopes (m) and associated *P* values that each one fits and the equations of curves. Download FIG S4, TIF file, 0.3 MB. Copyright © 2019 González-Torres et al. 2019 González-Torres et al. This content is distributed under the terms of the Creative Commons Attribution 4.0 International license.

### Role of HR in population structure and evolution.

**FIG 4:**
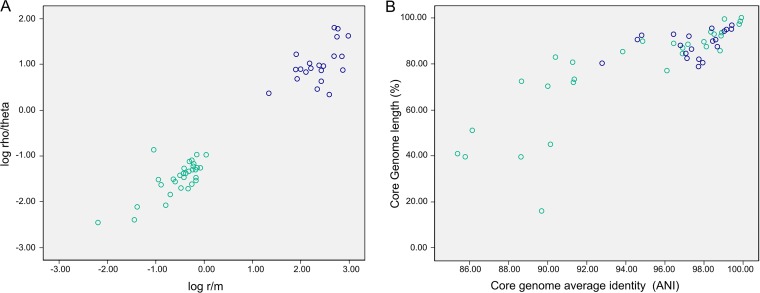
Influence of lifestyle and role of HR in population structure and evolution. (A) Correlation of r/m and rho/theta ratios (r/m of >1 [blue] or <1 [green]) for 54 species and (B) distribution based on core genome length and core genome identity (ANIb).

We explored the evolutionary impact of HR in the core genomes by assessing the relationship between the overall genome average nucleotide identity (ANI) based on BLAST (ANIb) and r/m and rho/theta ratios (where r/m is the ratio of probabilities that a given site was altered through recombination [r] and mutation [m] and measures how important the effect of recombination—relative to mutation—was in the diversification of the sample and rho/theta is the ratio of rates at which recombination [rho] and mutation [theta] occurred and is a measure of how often recombination events happen relative to mutations) (Fig. 4). Most species with positive logarithmic r/m values (r/m >0.33; blue dots in Fig. 4) showed ANIb values greater than 95% (Fig. 4), below which there was a significant fall in ANIb values and in the proportion of synthetic core genome regions. This value corresponds to the threshold observed in metagenomic studies for species with cohesive population structures (49–51). Comparisons between strains of the same species have revealed the same clusters and differences at ANIb values between 1% and 5% (5, 6), corresponding to a dominant evolutionary role for HR. In addition, 95% identity corresponds to a DNA-DNA hybridization value of 70%, above which two strains are considered to be from the same species and below which there is a sharp decrease in HR efficiency (14, 21, 52, 53). The average ratio of nonsynonymous substitutions to synonymous substitutions (dN/dS) across different lifestyles (Fig. S5 [additional file 2]) revealed that opportunistic pathogens and commensal species with large effective populations and high levels of HR, such as Escherichia coli (30), showed lower dN/dS values than obligate pathogens such as Chlamydia pneumoniae, even among species of the same genus. Although earlier observations of particular species (54–56) suggested this relationship, here we uncovered a general trend supporting a role of HR as the main evolutionary force—among other variables considered in this study. This important role of HR is predicted by the neutral model (10).

10.1128/mBio.02494-18.5 FIG S5 Correlation of rho/theta and horizontal gene transfer (HGT) content (A) and dN/dS average pairwise values amongst 54 species included in this study based on different lifestyles (B). Lifestyle distributions are represented with the following color code: black, endosymbionts and intracellular pathogens; blue, opportunistic pathogens; red, commensal and free-living pathogens; green, obligate pathogens. Download FIG S5, TIF file, 0.4 MB. Copyright © 2019 González-Torres et al. 2019 González-Torres et al. This content is distributed under the terms of the Creative Commons Attribution 4.0 International license.

### General model.

**FIG 5:**
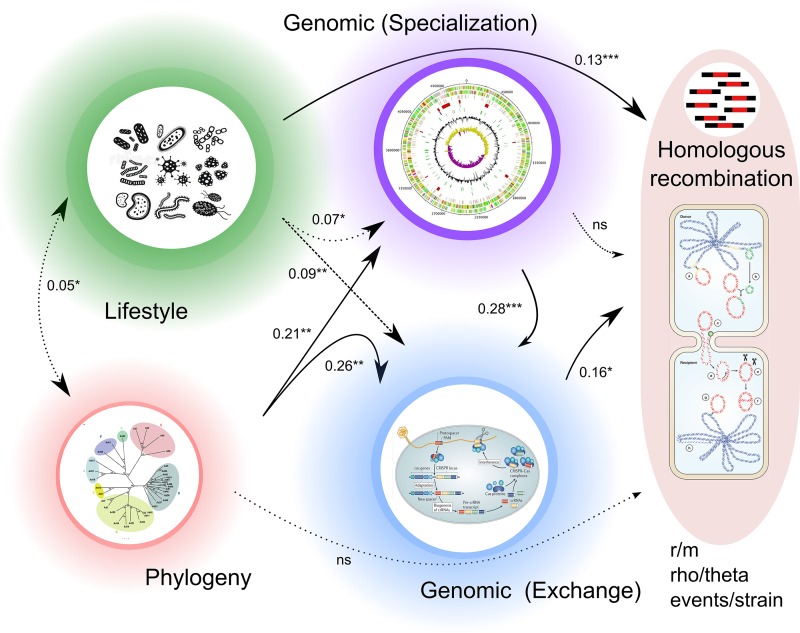
General model. A scheme is presented of a path analysis model proposed for the analysis of the influence of lifestyles, phylogeny, barrier/motility genomics variables, and genomics characteristics on HR levels detected among 54 species analyzed. Significant *r* values (*, *P* < 0.05; **, *P* < 0.01; ***, *P* < 0.001) for partial comparisons carried out during Mantel test are indicated. Arrows indicate relationships between variables, with the thickness of the arrows being proportional to the correlation between the connected variables.

We used general linear regression and path analysis to integrate all of the variables mentioned above and to study their relationships (see Materials and Methods). Lifestyle was the factor that best explained the distribution of HR in different species (27% of the variance; *P* < 0.05; linear model). Linear models with two variables showed that lifestyle in combination with other variables such as competence capabilities or the fraction of HGT (which alone explains 8% of the variance) can explain over 30% of the HR variation. We then carried out path analysis, which enables the investigation of direct and indirect interactions among variables. The resulting model (Fig. 5) confirmed that lifestyle had the largest direct effect on HR rates, together with variables related to motility and barriers. In addition, this model indicates that, as discussed above, phylogeny did not significantly affect HR levels in a direct way. However, this variable showed an indirect effect mediated through its influence on the genomic characteristics of the different species. Overall, ecological strategy and competence ability combined explained about 32% of the variance in HR events (*P* < 0.05, *r*^2^ = 0.317 [analysis of covariance {ANCOVA}, linear model]).

### Hot spots of gene exchange and adaptive implications.

**FIG 6:**
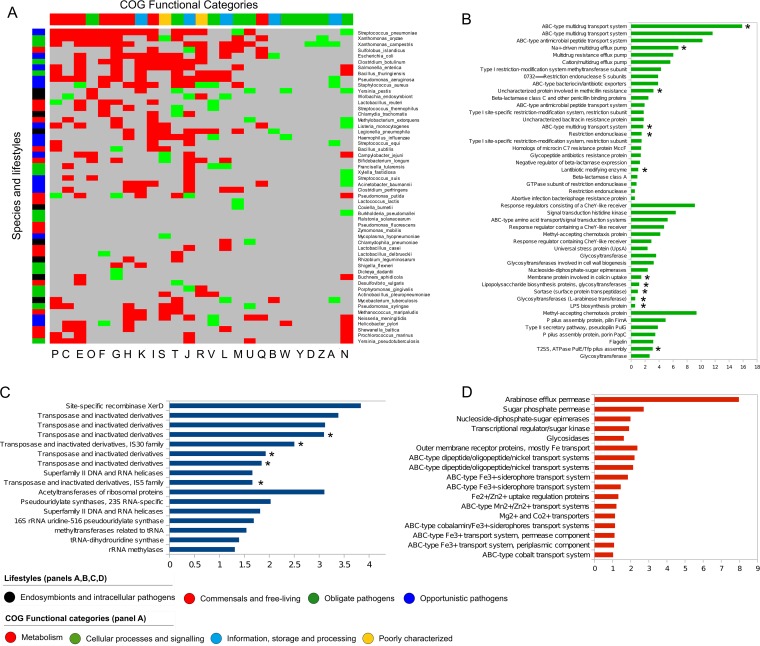
Gene flow and adaptive implications. (A) Heat map for similarity matrix representing the grouping of 54 species analyzed based on their profile and on those with significantly enriched (green) or underrepresented (red) genes (*P* < 0.05 [Fisher’s test; FDR correction, <0.1]). The *x* axis shows the layout of the functional categories and the *y* axis the species analyzed. (B to D) Distribution (%) of the most abundant GO terms among HR events and associated with (B) “Information, processing and storage and cellular processes” COG categories (red), (C) “Cellular processes and signaling” COG categories (dark blue), and (D) “Metabolism” COG categories (red). GO terms that presented significant enrichment or underrepresentation are marked with an asterisk (*) (*P* < 0.05, pFDR < 0.05 [Fisher’s exact test]).

Genes under positive selection or involved in interspecies genetic transfer have been found to be involved in adaptation to the environment (18, 57, 58). So far, however, we do not understand the possible functional implications of HR at the intraspecific level (16). To uncover prevalent genetic flows and their ecological significance in adaptation or HR processes, we explored the gene content and functional annotations that are overrepresented and underrepresented in HR events across species and lifestyles (Materials and Methods). These analyses revealed patterns (Fig. 6; see also Table S1E) which we explored through heat map and clustering analyses (Fig. 6). The identified clusters partially grouped some obligate and opportunistic pathogens as having similar functional patterns of exchanged genes as well as phylogenetically related species that share a lifestyle and have similar HR levels. Overall, the functional category that is most prevalent among exchanged genomic regions is “defense mechanisms” (Clusters of Orthologous Groups [COG] V), which was significantly enriched across all lifestyles. Genes in the “cell motility and secretion” category (COG N) were enriched among obligate and opportunistic pathogens, whereas the latter lifestyle also showed enrichment in “RNA processing and modification” (COG A) (*P* < 0.05 [false-discovery rate {FDR}, <10% [Fisher's test]) (Fig. 6). Notably, the majority of categories related to metabolic functions were underrepresented (Fig. 6). This is in stark contrast with what has been observed in interspecific exchanges, mostly mediated by illegitimate recombination, where metabolism always appears overrepresented (35). To obtain a more detailed insight into the possible functional implications of HR, we zoomed into the different categories and specifically looked at the lists of enriched terms and genes (Table S1E) in the other two main COG functional categories: “Information, processing and storage” and “cellular processes.”

Inspecting the “Information, processing and storage” COG categories, we identified a high abundance of terms connected with HR mechanisms and specific adaptive processes. We observed an overrepresentation among HR regions of the terms “serin recombinase XerD” (4% of enriched terms, the most abundant within enriched terms in the category COG L), and “transposable elements” (25%) (Fig. 6). These enrichments support the idea of a role of transposable elements and *XerD* in HR and integrative processes directly contributing to their own mobilization and likely that of hitchhiking neighboring genes (3, 30, 59). We observed reduced representation of genes related to transcription and protein translation and ribosomal structure (COG K and COG J), mainly in pathogens, while enrichments occurred in terms related to RNA processing (COG A) (Fig. 6). This is in agreement with predictions from the complexity hypothesis (60, 61), which states that extensive horizontal transfer mainly affects operational genes (those that are involved in housekeeping activities and are typically members of large, complex systems) whereas informational genes (those involved in transcription, translation, and related processes) are seldom horizontally transferred. Genes involved in HR within the COG K and COG J categories (8% of the total) were related to cell motility and chemotactic processes as well as encoding methylases, uridylases or rRNA and ribosomal protein acetylases. Of note, 2.5% of the genes associated with HR events were involved in rRNA and tRNA synthesis, which shows that the transfer of informational genes is not necessarily deleterious (6, 62–65). With respect to the enrichment of methylases, methylation of rRNAs has been linked to the acquisition of antibiotic resistance (66–68).

With respect to “cellular processes,” we noted that the most abundant or enriched terms were often directly related to resistance, pathogenicity, and adaptive mechanisms. For instance, we found significant enrichments (*P* < 0.05, positive false-discovery rate [pFDR] < 0.05 [Fisher’s exact test {FT}]) in multidrug or antibiotic resistance, transport systems, or beta-lactamic resistance, which also plays an important role in cellular communication processes or competition between strains of the same species (69–71). More precisely, we identified significant enrichments in type I RM systems, glycosyltransferases, and O-antigen clusters and virulence factors such as Fe transport systems or type II and IV transport systems. All of them are usually incorporated in the accessory genome (plasmids and GIs) and positively selected (72–76) and move from there to more stable genomic regions (75). Type II and IV transport systems are related to HR events in pathogens (77, 78); virulence capabilities and transferences mediated by transposons (79); and adaptation processes such as virus-host interactions, biofilm formation, secretion of virulence factors, adhesion to surfaces, and DNA transfer through conjugation (80). We found several enriched terms (*P* < 0.05, pFDR < 0.05 [Fisher’s exact test]), including glycosyltransferases and epimerases (involved in the synthesis and modification of surface elements [lipopolysaccharides, glycoproteins, and glycolipids]), O-antigen ligases, and lipopolysaccharide biosynthesis, corresponding to gene products that are usually encoded within O-antigen clusters present in GIs of free-living and pathogenic prokaryotes (45, 73, 81, 82). The role of O-antigen diversification in response to environmental viral pressure has been described in free-living organisms (83) as an adaptive mechanism of pathogens to evade immune system (84, 85) or mediating the intraspecific interaction responses in core bank species (71). As for the enrichment in Fe transport systems, most of Fe transporters contribute to environmental adaptation under iron-limiting conditions in both free-living and pathogenic human bacteria (18, 59, 81). Among terms related to “cell process and signaling,” the main terms inside “signal transduction” (COG T) involved chemotactic processes, regulation, and universal stress of union gene (UpsA). Taken together, the results of functional analysis of genes enriched in HR regions uncovered enrichments of functions related to important known adaptive pressures and point to a role of selection in shaping patterns of HR.

## DISCUSSION

Our comprehensive analysis covering 54 diverse species and using a common methodological framework provides an overall view of the patterns of intraspecific HR in prokaryotes and does so at unprecedented levels of scale and resolution. The validity of our approach is underscored by the congruence of our results with some smaller scale analyses focused on some of the species included in our analysis. However, besides corroborating earlier observations, the broader scope of our approach allows us to unveil global patterns and test trends predicted by different theoretical proposals. In addition, we provide a general model of the interrelationships of the different genomic and ecological factors studied. Overall, our analysis reveals that intraspecific HR is pervasive and occurs in all analyzed species with various levels of impact. After correction for genome size and number of strains was performed, the data revealed that the fraction of the genome affected by HR ranges from 0.5% and 4.13 events/strain (in Yersinia pestis) to more than 33.74% and 152.86 events/strain (in Streptococcus pneumoniae).

We analyzed a range of genomic and ecological factors that may influence these differences. Among all the factors analyzed, lifestyle emerges as the most relevant, accounting for up to 27% of the variability in HR levels. This explains earlier observations of important differences in HR rates between species of the same genus with different lifestyles (55) and even among lineages of the same species (16, 86). Differences in HR levels between obligate and opportunistic pathogens may relate to differences in the degree of host specialization and in environmental conditions, which is more variable in opportunistic pathogens (13, 42). For instance, we detected the largest HR levels in species such as S. pneumoniae and Helicobacter pylori, for which our study included strains involved in the same polyclonal infection processes (31, 87). These high levels of HR in pathogens may relate to the selection pressure imposed by the host's immune system to increase the antigenic variability, which has been shown to activate recombination systems and induction of competition and repair systems (44, 75). In this sense, and in addition to having a role in maintaining the cohesion of population clusters, HR can participate in adaptive processes as indicated by the functional analysis of exchanged genes, highlighting the presence of enriched terms directly related to resistance and pathogenicity mechanisms and adaptive processes.

Genomic factors explained a significant part of the observed variability and revealed two clearly differentiated patterns. At one extreme, promiscuous species with large genome sizes and accessory genomes presented higher HR rates and high competence capabilities and presented stronger regulatory mechanisms to control genetic exchange (RM systems and CRISPR-Cas). These promiscuous species tended to be associated with free-living or opportunistic pathogenic lifestyles. At the opposite extreme, we found less-promiscuous species with reduced genomes and low rates of HR and with opposite trends with respect to the aforementioned genetic elements. These species were typically endosymbionts, intracellular pathogens, or obligate pathogens.

Our findings support the idea of a role of competence mechanisms in the exchange of smaller HR events. In contrast, and in accordance with previous suggestions based on individual observations (3, 16, 32) the presence of large recombination events, although uncommon, tends to be associated with the presence of conjugation mechanisms and positive selection. Competence has been considered an adaptive strategy that accelerates adaptation of natural transformants (44, 48, 88). Importantly, we observed that an increase in CRISPR-Cas system type content correlated with a higher proportion of short event fragments among the competent species, supporting the idea of coevolution of the two mechanisms. Finally, we note a general correlation between *rec* gene content and RM systems such as has been previously observed in a limited number of promiscuous species (19, 46, 47) in which the entry of heterologous genetic material and the increase in genome size justify greater control and selection, in accordance with the proteomics constriction theory (38, 89). The greater level of diversity was found in the type III MR system prevalent in those groups (see Fig. S3 in the supplemental material), which showed higher rates of HR and genome accessory content (Fig. 2 and 3).

Taken together, our analyses provide an overall picture of the different ecological, evolutionary, and genomic factors that modulate the impact of bacterial HR, clarifying the relative importance of each. Although some of the correlations found could be considered to have been expected because they are compatible with current biological knowledge, this should not blur the importance of the finding. First, we have tested many different factors, all of which have been previously proposed to be relevant for HR in the light of current biological knowledge and thus could have been expected to show positive correlations *a priori*. However, our data have not shown support for all of them and, among those that our data have supported, the relative weights are different. For instance, our results support the idea of a stronger role of lifestyle than of phylogenetic background in determining the extent of recombination. The opposite finding—that recombination rates are more similar within a genus or clade regardless of lifestyle—would also make sense from a biological standpoint. Such a finding would simply suggest that recombination rates are more constrained evolutionarily. Thus, our findings are important to define how different factors and processes are weighed in modulating recombination.

Finally, considering their strong relationships with the analyzed ecological, functional, and genomic variables, our data support the idea that HR is one of the main evolutionary mechanisms shaping prokaryotic core genomes. In this regard, our study uncovered many functional links between exchanged genes and specific ecological adaptations, providing the basis of further research. For instance, we observed an enrichment of O-antigen genes among recombined regions, and the diversity of O-antigens is known to determine susceptibility to bacteriophages (90). It could be hypothesized from this that recombination involving these genes may favor adaptation to pressure exerted by phages. Such a hypothesis could be tested, for instance, by *in vitro* evolution experiments exposing mixed populations of bacterial strains to particular phages, whereby recombination in regions encoding O-antigens would support the idea of a selective advantage for some of the bacterial strains. Construction of a detailed list of testable hypotheses that could be derived from our functional enrichments is beyond the scope of this work. Further scrutiny by experts examining the different proteins and species will certainly lead to additional specific and relevant testable predictions.

As we have seen, HR provides a mechanism through which genes or variants can be exchanged, thus providing a substrate upon which selection can act. At the same time, HR acts as a cohesive force keeping population structures intact. In accordance with this role, and extensively supporting the neutral model (10) mentioned above, the species with over 95% ANIb presented r/m values above 0.25, a value that is considered to represent the minimum threshold at which HR acts as a cohesive force on the emerging population clusters. Although, at first glance, support for the neutral model may seem at odds with the reported footprints of selection, this is not necessarily so. As the authors of the neutral model (10) stated, “the use of neutral models of mutation and drift is not a denial of selection, but a recognition that much observed population genetic structure can be explained in simple terms.” Thus, the recombination would occur stochastically and would result in the emergence of population clusters even in the absence of selection. However, due to differences in fitness between the different recombined genomes in a given environment, selection would act to determine which population clusters, and which individuals within each cluster, are more likely to survive.

## MATERIALS AND METHODS

### Genome data set.

Bacterial and archaeal genomes fully assembled into a single superscaffold were downloaded from the NCBI FTP site (https://www.ncbi.nlm.nih.gov/genomes). We considered only species for which three or more fully sequenced genomes of different strains were available. This resulted in a total of 338 genomes (325 bacterial genomes and 13 archaeal genomes) from 54 species, each including 3 to 15 genomes (Fig. 1). Additionally, 19 genomes from strains belonging to the same genus but not the same species were selected and included in five control groups (16S ANI, <98.7%) (22) that were used to estimate false-positive rates in HR detection methods (see below).

We validated the fitting of each clade of selected strains to the 16S divergence criterion to define species using rRNA 16S pairwise alignments using SILVA Incremental Aligner (SINA) 1.2.11v (91), ensuring that the average nucleotide identity (ANI) within the 16S sequence was within the species range (similarity of >98.7%) (21) or, in the case of the controls, within the genus range only (similarity of <98.7%) (21). Species were classified into the following four main groups of lifestyles, as defined previously (13): (i) intracellular pathogens and symbionts, (ii) opportunistic pathogens, (iii) obligate pathogens, and (iv) free-living microbes.

### Whole-genome alignments: positional orthologs and values corresponding to genome-wide average nucleotide identity based on BLAST (ANIb).

The genomes of the species were aligned using the progressive function from the Mauve Package 2.3.1v using default parameters (92). The output consisted of the alignment map showing the local colinear blocks (LBCs) and the main rearrangements and of the sequences contained in the aligned blocks (eXtended Multi-FastA [*xmfa] format) and the positional orthologs therein (*ort files). The output files were parsed with a python script which selected the core genome (genes shared among all the strains) and accessory genomes (genes missing in some strains) (93, 94).

### Genome-wide average nucleotide identity based on BLAST (ANIb) between strains within species.

The genomic sequences within each species were compared in a pairwise mode using the Nucmer function from the MUMmer package v3.0 (95). The resulting coordinate files (*coords files) were parsed with a python script to calculate the percentage of identity for the aligned sequences. For this, fragments that overlapped over 10% of their length were joined, and those contained within a larger fragment were filtered out. An overall nucleotide identity value based on BLAST (ANIb) was obtained for each comparison as the average of each aligned fragment weighted by its size.

### Detection and characterization of recombination events.

RDP4 v4.15 (24) was used to detect and characterize recombination events. This program implements different recombination detection methods to both detect and characterize the recombination events that are evident within a sequence alignment without any prior user indication of a nonrecombinant set of reference sequences. The implemented algorithms use a combination of methods based on partitioning schemes, dynamic scanning window strategy-based algorithms, and testing schemes that generally consist of two steps of analysis, the first performed to detect changes in the phylogenetic sequence relationships between partitions and the second to statistically test the approximate significance of these changes. Genomic alignments obtained via the *xmfa format described above were used as input files. Recombination events were predicted in a two-step procedure. In the first, exploratory phase, the following four different methods were run: RDP (96), GENECONV (97), MaxCHI (98), and Chimera (99). In the second phase, the program rescanned every detected event more thoroughly with the RDP, GENECONV, MaxCHI, Chimera, and 3Seq algorithms (100). The window size settings for the RDP algorithm was adjusted to 90 nucleotides, and the number of variable sites per window was set to 210 in the case of MaxCHI. For the remaining methods, default parameters were applied. In all cases, a cutoff probability (*P*) value of <0.001 was used. Only recombination signals detected by at least three of the five methods were considered. Finally, the predicted recombination events were manually curated, and the breakpoints were inferred using the MaxCHI method (which is considered the most accurate breakpoint detection method among the five nonparametric methods implemented in RDP3) (24). The following variables were recorded for each comparison: fraction of genome recombined, number of recombination events per strain, and distribution of the sizes of the recombination events. To obtain a rarefaction curve in terms of the number of HR events detected depending on the number of compared genomes, we repeated the analysis with subsets of 10 randomly selected E. coli strains from our set.

### Experimental design and variables.

We evaluated several genomic, evolutionary, and ecological variables obtained from different databases (see Table S1E in the supplemental material). Genomic variables included the following: (i) size of core genome (number of kilobases and number of genes); (ii) size of GIs; (iii) number of ribosomal operons; (iv) tRNA content; (v) presence of elements that favor intraspecific DNA exchange (also known as motility variables) such as content of RM systems (type I, II, II, and IV) and competence capabilities (*com* gene content); and (vi) presence of elements that make interspecific DNA exchange difficult (i.e. barriers) such as CRISPR-Cas system content. Evolutionary variables included the following: (i) phylogenetic position; (ii) ANIb; (iii) ratio of nonsynonymous substitutions to synonymous substitutions (dN/dS); (iv) number of recombinant events by strain (events/strain); (v) recombination/mutation ratio (r/m); and (vi) proportion of recombined genome. The relevant qualitative ecological variables included the following: competence capabilities and lifestyle classification from previous MLSA studies (13, 16) and JGI database metadata assigning each set of strains to one of the four main groups mentioned above.

### Functional analysis.

Functional annotations were retrieved for all 338 strains from the Integrate Microbial Genomes (IMG) database (Joint Genomics Institute) (101). Sequences of the predicted recombined fragments were retrieved from the genome FASTA files using the positional coordinates provided by RDP4 v4.15 (24). For those sequences, *de novo* gene prediction was performed employing the Integrative Microbial Genomes (IMG) system (Joint Genomics Institute) (101). Recombinant regions shorter than 10 kb were annotated with an algorithm implemented for metagenomic data (IMG/M ER), and a genomic algorithm (IMG ER) (101) was used for those longer than 10 kb. Functional terms from the Clusters of Orthologous Groups (COG) (102) and Gene Ontology (GO) (103) databases were retrieved for both genome and recombination fragments using the JGI platform and Blast2GO (B2G) software (104, 105), respectively. In the latter case, hits with an E value lower than 1 × 10^−20^ and amino acid sequence identity higher than 55% were considered. Finally, KEGG EC numbers (106) were retrieved.

### Statistical analyses.

Tests for functional enrichment of genes contained in recombination fragments versus the genomic background were performed using Fisher’s exact test (FT) with the COG and GO annotation terms. The Fisher’s test was performed for each of the functional categories in each species by applying a false-discovery rate (FDR) correction and designating 0.05 the *P* value threshold for over- or underrepresentations, as implemented in the Gossip tool from Blast2Go. The distributions of the variables described above were compared using Kruskal-Wallis/Jonckheere-Terpstra tests as implemented in the SPSSv22 statistical package complemented by multiple comparisons or Bonferroni adjustment.

Bivariate and partial correlations were used to explore relationships between quantitative variables using the SPSSv22 statistical package. For this purpose, parametric and nonparametric tests were run using Pearson and Spearman correlation coefficients, respectively, to assess bilateral significance (marginally significant, <0.1; *, *P* < 0.05; **, *P* < 0.01; ***, *P* < 0.001). Mantel-Haenszel and partial Mantel-Haenszel tests were applied to Euclidean distance arrays generated from related recombinant variables (events/strain and proportion of genome recombined), motility variables (competence and RM content), barrier variables (CRISPR-Cas and HGT), and phylogeny genomic and lifestyle class. *P* values of less than 0.05 were considered significant. For those factors involved in the distribution of the variable homologous recombination data, a general model was built from the results of the Mantel-Haenszel test by means of a path analysis.

### Evolutionary genomics analysis.

We reconstructed the clonal genealogy using ClonalFrame 1.2v (27) and the genomic alignments sorted with MAUVE 2.3.1v as input. Core alignments were extracted by keeping only those regions that were aligned for all genomes over at least 500 bp. Three independent ClonalFrame runs were performed, each consisting of 40,000 iterations. The first half of these iterations was discarded as representative of Markov chain Monte Carlo (MCMC) burn-in. The convergence of the tree runs was checked by manual comparison, making sure that they produced consistent estimates of the clonal genealogy and of the global parameters r/m (where r = rate of recombination and m = rate of mutation) (13) and rho/theta (σ/θ) (107) (where σ and θ are the rates of occurrence of recombination and mutation, respectively) with 95% credibility. The σ/θ ratio is a measure of the frequency at which recombination occurs relative to mutation, and the r/m ratio is a measure of the rates at which nucleotides become substituted as a result of recombination or mutation and estimates the relative effects of HR on genetic diversification of populations.

The amino acid sequences for the positional orthologous genes were retrieved from the GenBank files. Pairwise sequence alignments between selected strains of each species were performed with MUSCLE 3.8v (108). The resulting alignments were reverse translated to codon-based nucleotide alignments using trimAL v1.3 (109) and the corresponding coding sequences. Finally, dN/dS values were obtained using the CodeML function (pairwise mode with model 1 nonsynonymous [NS] sites [0 parameters]) of PAML package 4.4v (110).

### Ethics approval and consent to participate.

No data from humans were used in the work described in this article.
