# Supplementary figures and images for: Impact of Homologous Recombination on the Evolution of Prokaryotic Core Genomes
Source: mBio. 2019 Jan 22;10(1):e02494-18. doi: 10.1128/mBio.02494-18 (PMC6343036; doi:10.1128/mBio.02494-18)

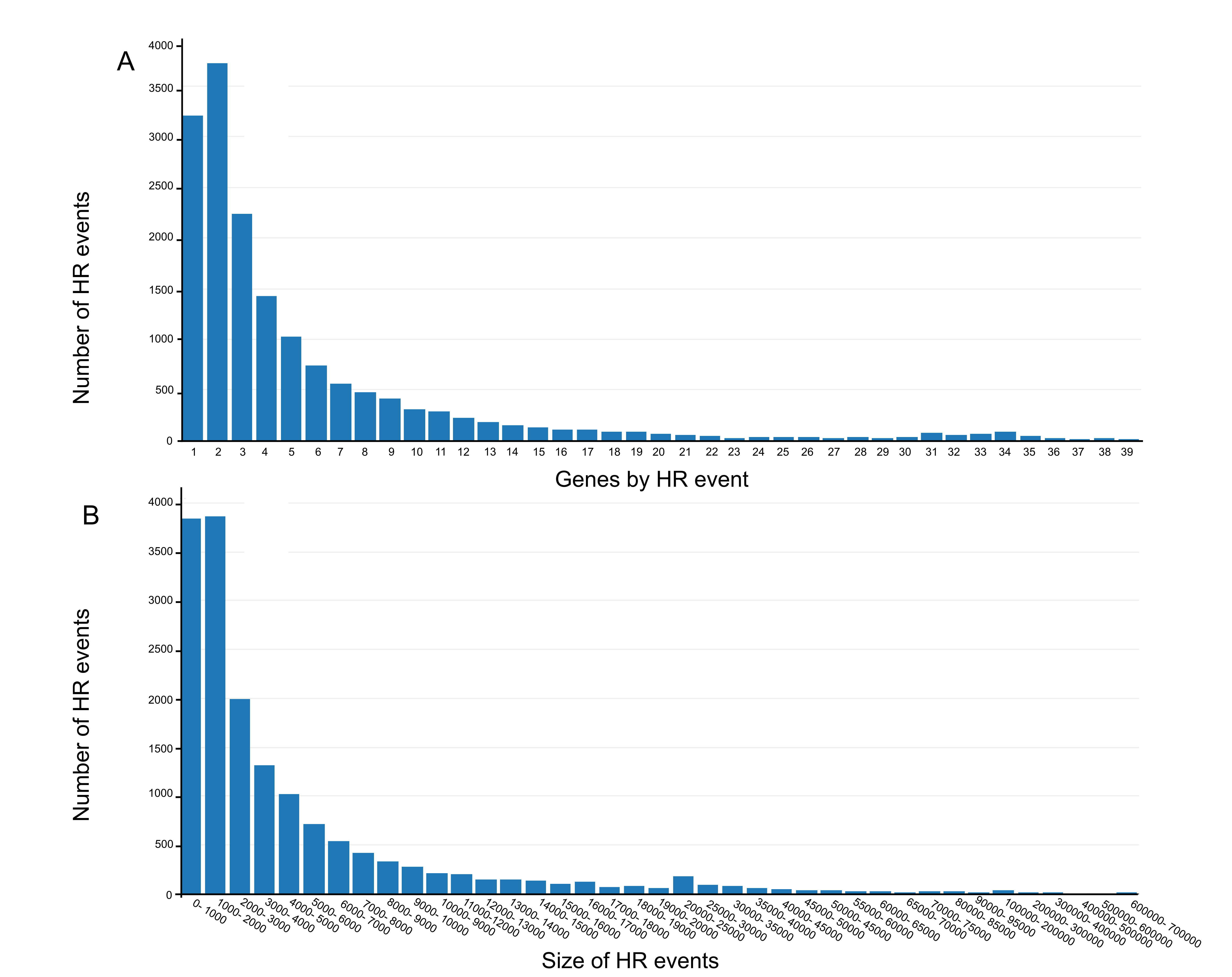

Supplement: FIG S1 [file mBio.02494-18-sf001.tif]

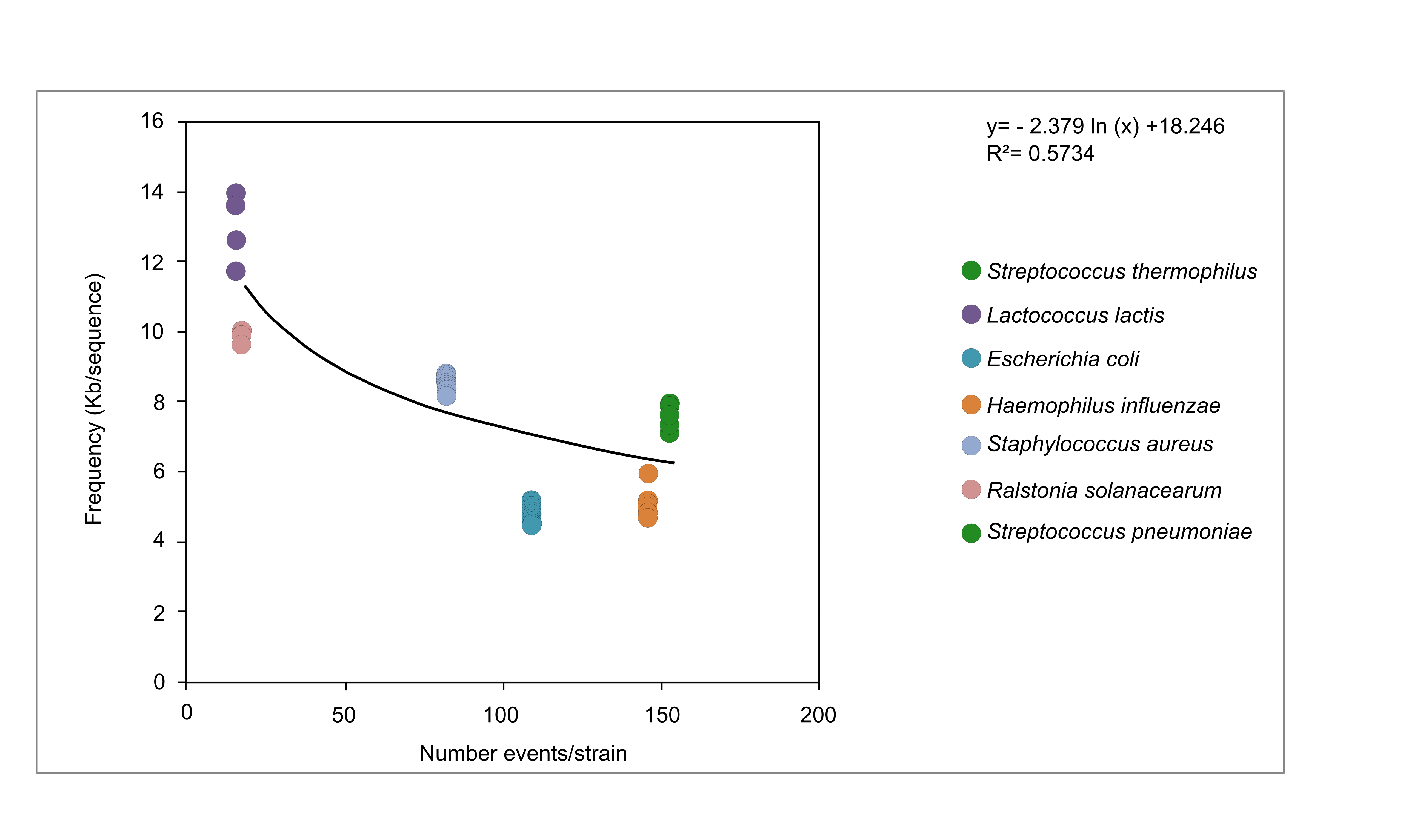

Supplement: FIG S2 [file mBio.02494-18-sf002.tif]

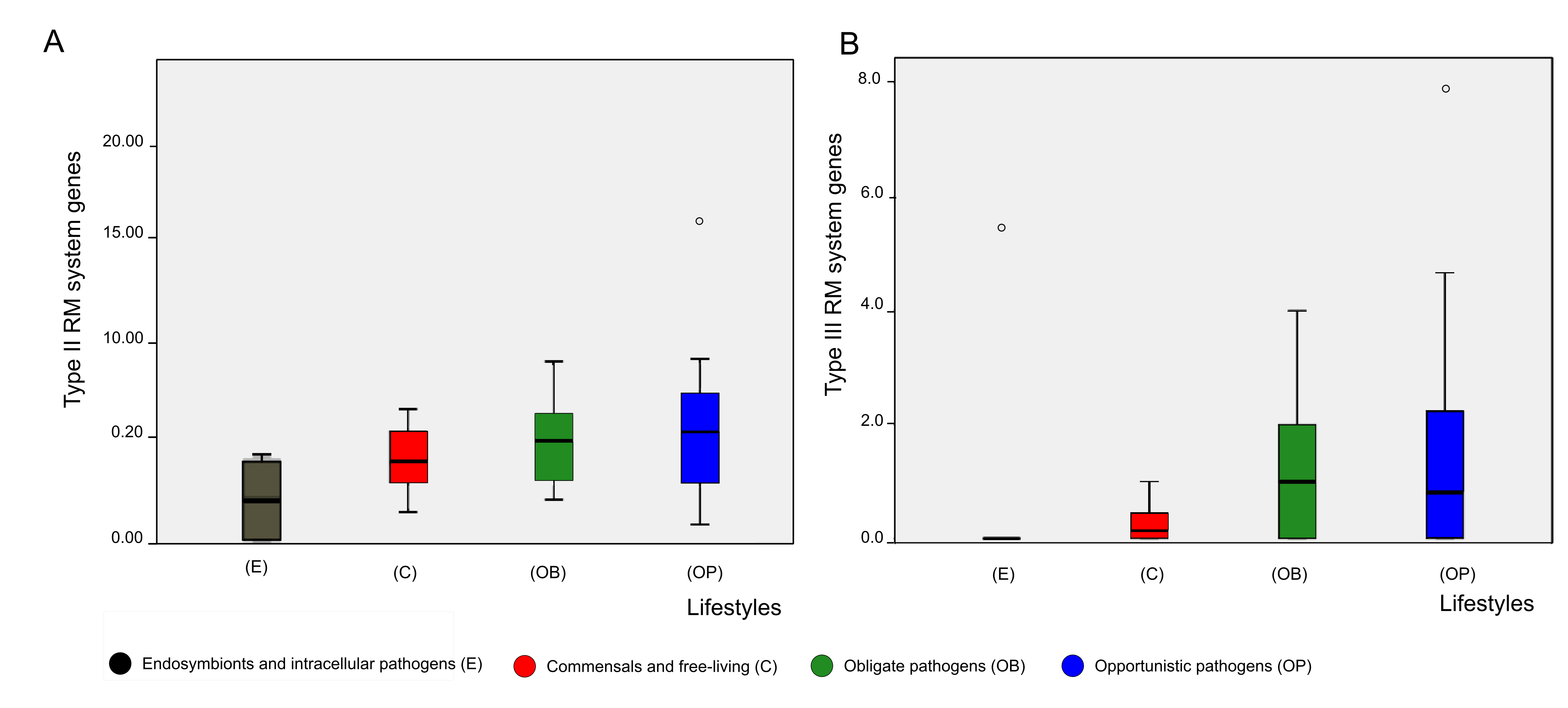

Supplement: FIG S3 [file mBio.02494-18-sf003.tif]

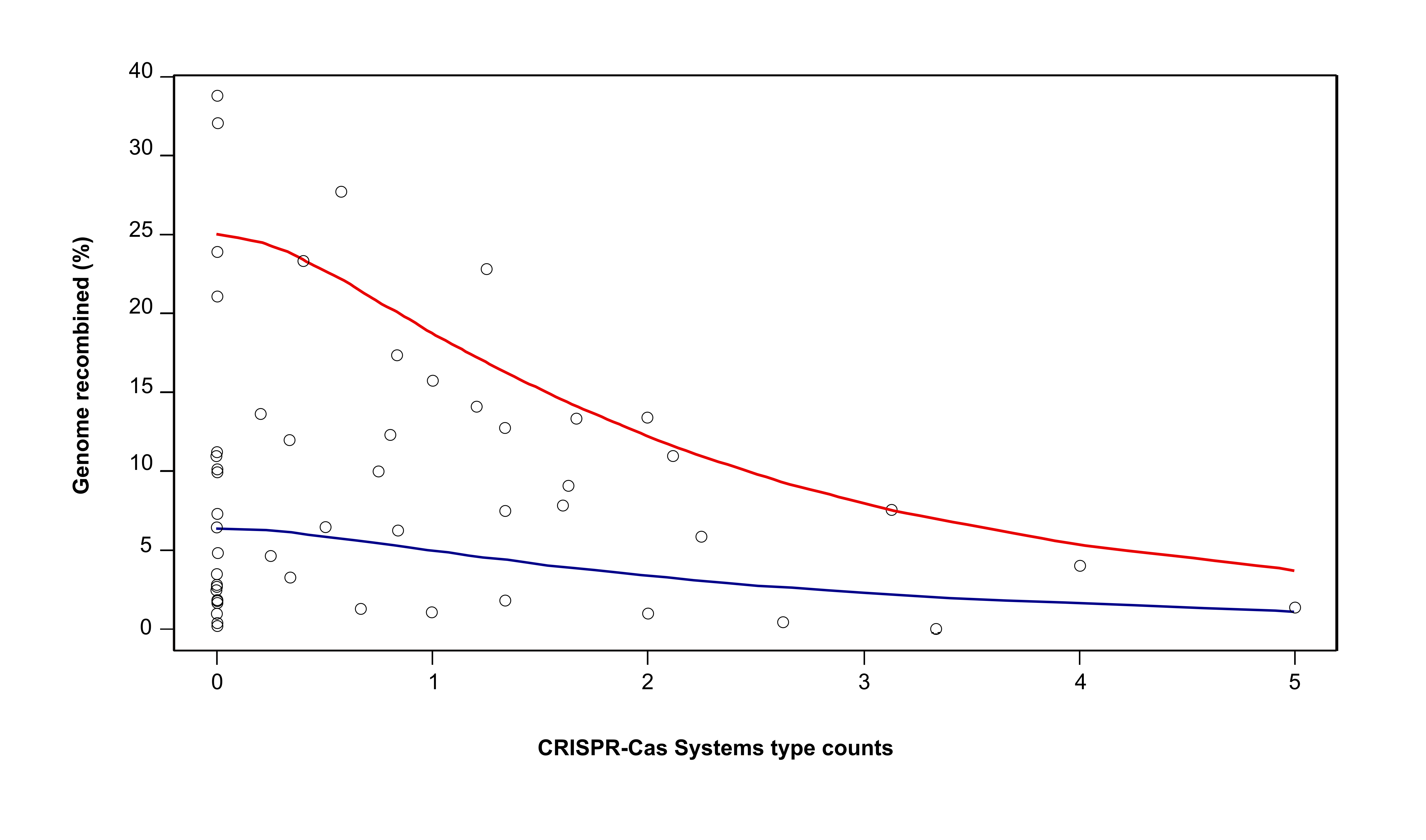

Supplement: FIG S4 [file mBio.02494-18-sf004.tif]

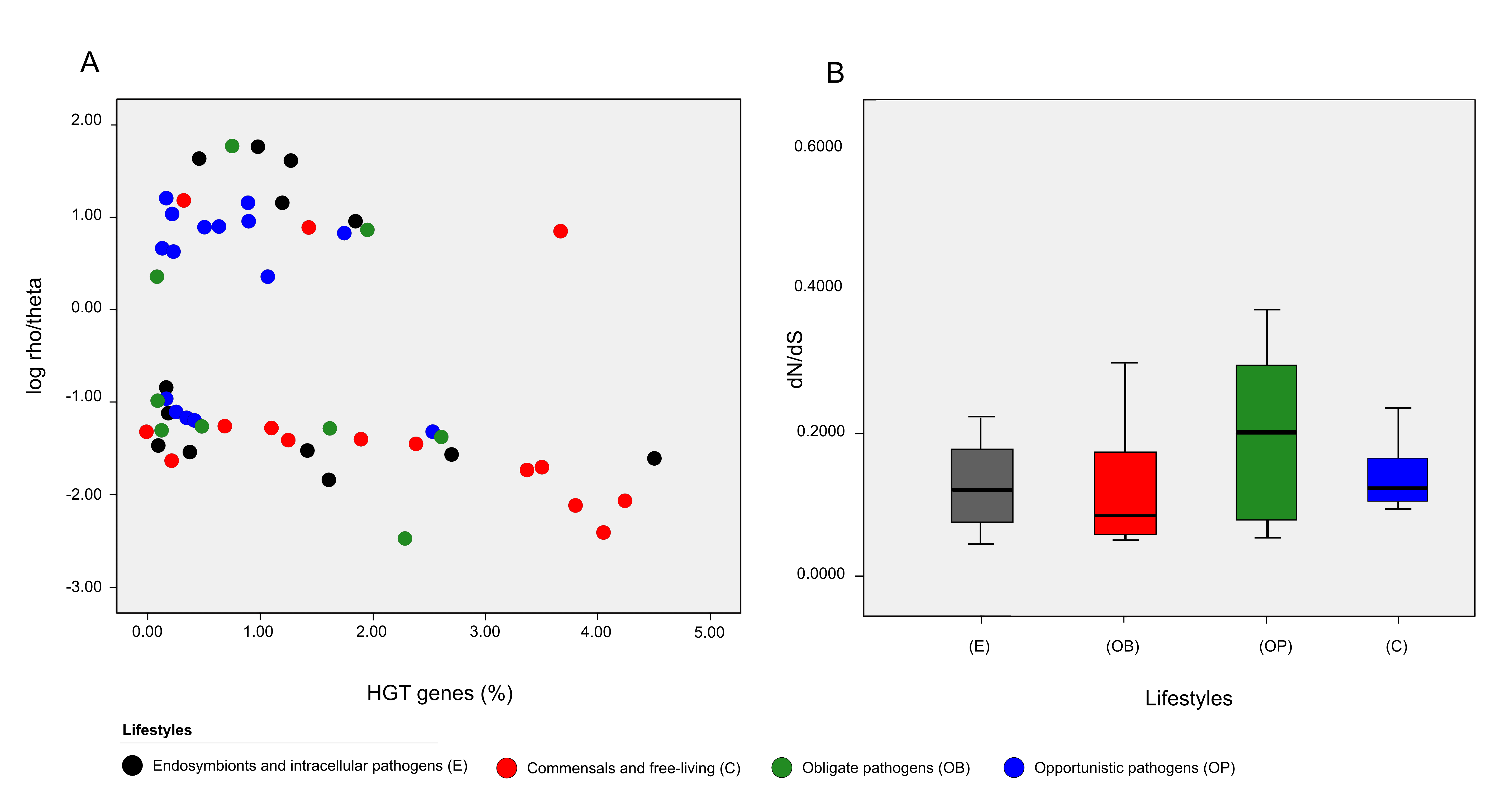

Supplement: FIG S5 [file mBio.02494-18-sf005.tif]
